# Percutaneous nephrolithotomy in supine position with less than 24-hour hospital stay; a single-center experience

**DOI:** 10.1080/2090598X.2023.2234254

**Published:** 2023-07-16

**Authors:** Morshed Salah, Bela Tallai, Tawiz Gul, Omar Aboumarzouk, Maged Alrayashi, Mohamed Abdelkareem, Hatem Kamkoum, Mohammed Ibrahim, Mohammed Ebrahim, Hossameldin Alnawasra, Salvan Alhabash, Ahmed Ismail, Maged Alghashmi, Abdulla Al-Ansari

**Affiliations:** a Urology Section, Surgery Department, Hazm Mebaireek General Hospital, Hamad Medical Corporation, Doha, Qatar; b College of Medicine, Qatar University, Doha, Qatar; c Urology Department, Hamad General Hospital, Hamad Medical Corporation, Doha, Qatar; d Anesthesiology Section, Hazm Mebaireek General Hospital, Hamad Medical Corporation, Doha, Qatar; e Weill Cornell Medicine-Qatar, Doha, Qatar

**Keywords:** Day care, ambulatory, percutaneous, nephrolithotomy, PCNL

## Abstract

**Objectives:**

To report our initial experience of day care percutaneous nephrolithotomy (PCNL) with early hospital discharge within less than 24 hours of the procedure.

**Patients and Methods:**

The files of patients treated with PCNL between 1st January 2020 till 31st December 2022 were retrospectively reviewed. Day care PCNL was defined as the discharge of patients either on the same day or within 24 hours after surgery. Patient age, ASA score, body mass index, stone diameter, laterality, stone burden, Hounsfield unit, and Guy’s score were analyzed. Operative time, size of the access tract, method of lithotripsy, estimated blood loss, and length of hospital stay were also recorded. Postoperative complications were stratified according to the Dindo-Clavien classification. The primary outcome was to evaluate the feasibility and safety of early discharge within 24 hours after PCNL compared to the in-patients who were kept in hospital for at least 2 days after surgery.

**Results:**

A total of 85 patients underwent PCNL at our center of whom 36 patients were discharged within 24 hours (day care PCNL) of the procedure and 49 patients were kept for at least 2 days (in-patient PCNL). In the day care group, median stone burden was 465 mm2 (360-980) and 18 patients (50%) had Guy’s stone score ≥ III. The median tract size was 24 (13-30) and endoscopic combined intrarenal surgery (ECIRS) was performed in 7 cases in the day care group. Tubeless PCNL was carried out in 88.8% of the day care surgery group compared to 37.5% in the in-patient group (p < 0.0001). The postoperative complication rate was comparable between both groups (13.8% vs 22.4% for day care vs in-patient group, respectively, p = 0.08).

**Conclusions:**

Day care PCNL is feasible and safe for selected patients including those having large stone burden without increasing the risk of complications or readmission rate.

## Introduction

Percutaneous nephrolithotomy (PCNL) has revolutionized renal stone surgery since its introduction by Fernstrom and colleagues [1]. PCNL is considered the gold standard for the management of renal stones larger than 2 cm [2,3]. The last decade has witnessed huge modifications in PCNL techniques to achieve higher stone clearance rate and minimizing the morbidity of the procedure. These modifications included, for example, miniaturization of the equipment, refinement of renal access dilation tools, methods of intracorporeal lithotripsy including modern laser technology, and simultaneous use of flexible ureteroscopy with PCNL to reduce the number of access tracts in complex cases [4–6].

The incidence of perioperative complications following PCNL ranges between 12% and 14% in recent series [7,8]. However, majority of these complications are low grade and can be treated conservatively. Only small percentage of patients need hospital readmission [9]. These findings, together with the refinements in the surgical techniques, motivated the endourologists to explore the feasibility of early patient discharge within the first 24 hours after PCNL, namely, day care PCNL or ambulatory PCNL. Early discharge has several advantages for the patient like encouragement of early mobilization, early return to daily activities and reduction of nosocomial infections. Moreover, reduction of the cost of the procedure and increased hospital bed turnover would benefit the health care system [10].

In this article, we thought to report our initial experience of day care PCNL with early hospital discharge less than 24 hours of the procedure. We aim to assess the safety and feasibility of early hospital discharge and compare its complications and readmission rates with the standard PCNL.

## Patient and methods

### Study design

After institutional review board approval, the electronic files of patients treated with PCNL in a tertiary referral center between 1 January 2020 and 31 December 2022 were retrospectively reviewed.

### Patient population

**Figure 1.**
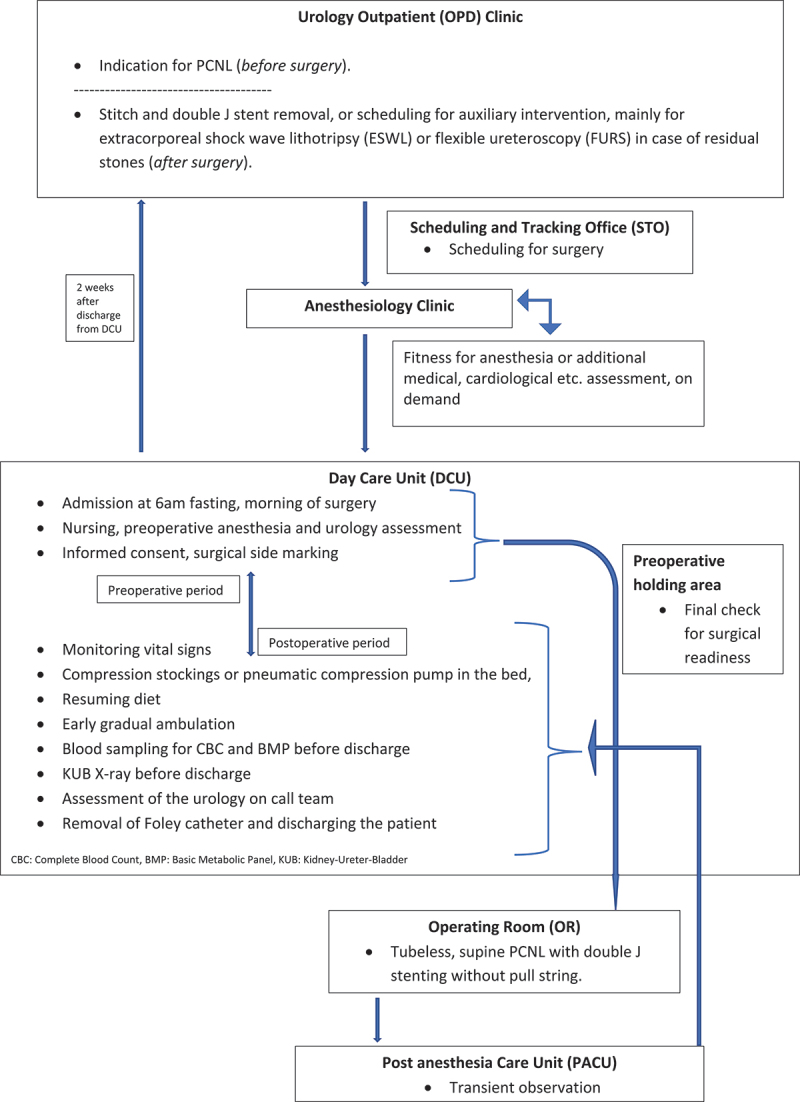
Patient flowchart for day care PCNL.

A protocol for day care PCNL was well established and refined by the urology section of our hospital. Day care PCNL was defined as the discharge of patients either on the same day or within 24 hours after surgery. Our detailed patient pathway algorithm for day care PCNL is demonstrated on Figure 1. All patients were admitted to our day care unit (DCU) electively at 6 o’clock in the morning fasting from midnight. All patients were counselled preoperatively about the possibility of early discharge from the hospital within 24 hours after the procedure, and a written informed consent was signed by the patient and obtained.

Eligibility condition for discharging patients home within 24 hours after the end of surgery are summarized in (Table 1). Those patients who met the eligibility criteria for the day care surgery were kept in the DCU till discharge. Exclusion criteria for day care surgery included: solitary kidney, transplant kidney, chronic kidney disease (CKD) with estimated GFR less than 60 ml/min, associated congenital anomalies or malformations and patient age less than 16 years.

**Table 1.** Eligibility criteria for day care PCNL.

Negative urine culture and normal kidney function within 1 month before surgery. Uncomplicated PCNL. Vitally stable, conscious, oriented, afebrile patient in the postoperative period. Numeric pain score <3 out of 10. Postoperative white blood cell count <15 million/ml and hematocrit within the normal range. Clear urine output via Foley catheter and dry dressing. Proper position of double J stent on postoperative KUB X-ray. Negative physical examination before discharge. Motivated patient, acceptance, and agreement for early discharge. Easy access to the healthcare system.

Demographic data including patient age, associated comorbidities, ASA score, body mass index (BMI), stone diameter, laterality, stone burden, and Hounsfield unit (HU) were analyzed. Complexity of the stone was assessed using the Guy’s score. Operative parameters including type of anesthesia, operative time, size of the access tract, method of lithotripsy, estimated blood loss (EBL), and length of hospital stay were also recorded. Postoperative complications were documented and stratified according to the modified Dindo-Clavien classification. The length of hospital stay was calculated in hours as the time interval between the end of surgery till the time of discharge order in the electronic patient’s data recording system.

### Surgical technique

A single 1.5-g intravenous Cefuroxime antibiotic prophylaxis was given upon induction of anesthesia based on our local antibiotic policy. Patients received either general or spinal anesthesia. A balloon tipped 6-F ureteric catheter was advanced into the renal pelvis under cystoscopic guidance to opacify the renal collecting system using contrast. Renal punctures were performed under fluoroscopic guidance after selecting the most appropriate calyx. A 0.089-cm flexible tip guidewire was inserted through the needle sheath into the collecting system. Tract dilatation was performed using fascial or balloon dilators according to surgeon’s preference. The size of the access tract varied from 13F to 30F according to stone size and surgeon’s preference.

Stone fragmentation was accomplished using Thulium fiber laser or combined electromagnetic and pneumatic lithotripter EMS LithoClast® Trilogy or Olympus Shock Pulse-SE depending on the size of the access tract. At the end of the procedure, the patient’s stone-free status (SFS) was assessed by X-ray film. A double J ureteric stent was left in situ in all procedures. The operative time was calculated from ureteric catheter insertion to completion of the intervention. A nephrostomy tube was placed in addition to double J stent in selected patients according to surgeon’s recommendation and was removed before discharge.

All the procedures were carried out in supine position by experienced endourologists or endourology fellows under direct supervision by experienced surgeons. Single tract was performed in all patients, and simultaneous flexible ureteroscopy was performed in cases of complex or staghorn stone to avoid multiple punctures.

### Follow up

Patients were counselled again about the necessary behavior and precautions for home and a written instruction was also given to them before discharge including to call for an ambulance when fever/chills, intolerable flank pain, spontaneous bloody urine, urine- or bloody leak at the wound experienced at their accommodation. After 2 weeks, control X-ray film or spiral CT scan was carried out to assess SFS before removal of the double J stent.

### Study outcomes

The primary outcome was to evaluate the feasibility and safety of early discharge within 24 hours after PCNL compared to the in-patient group who were kept in hospital for at least 2 days after surgery.

### Statistical analysis

For categorical variables, Chi-square and Fisher’s exact tests were used for comparison whenever appropriate. Differences in continuous variables were assessed using the Mann–Whitney U-tests and paired sample t test according to the distribution pattern. The statistical analysis was conducted using IBM v. 20 statistical software with *P* -value < .05 was considered statistically significant.

## Results

During the specified period, a total of 85 patients underwent PCNL at our center, of whom 49 patients were eligible for day care surgery. We had 13 frail patients with poor socio-intellectual status, who were not able to leave the hospital because of personal factors despite being eligible for discharge. After exclusions, a total of 36 patients were discharged within 24 hours (day care PCNL) of the procedure and 49 patients were kept for at least 2 days (in-patient PCNL). Baseline demographics of both groups are shown in Table 2. No significant difference was noted between both groups apart from stone diameter, which was significantly smaller in the day care group (34.7 vs 43.5 mm, *p* = 0.004). In the day care group, median stone burden was 465 mm^2^ (360–980) and 18 patients (50%) had Guy’s stone score ≥ III. The median tract size was 24 (13–30), and endoscopic combined intrarenal surgery (ECIRS) was performed in 7 cases in the day care group. Tubeless PCNL was carried out in 88.8% of the day care surgery group compared to 37.5% in the in-patient group (*p* < 0.0001) (Table 3).

**Table 2.** Baseline characteristics of the 85 patients treated as either day care or in-patient PCNL between January 2020 and December 2022.

	Day care PCNL	In-patient PCNL	P
Age (Mean ±SD)	41 ± 7.4	43 ± 7.9	0.1
BMI (kg/m^2^, Mean ±SD)	26 ± 3.8	26 ± 3.9	
ASA score			0.1
I	26 (80.6%)	31 (63.3%)	
II-III	6 (16.7%)	18 (36.7%)	
Stone diameter (mm, Mean ±SD)	34.7 ± 11.1	43.5 ± 14.7	0.004
Stone burden (mm^2^, median, IQR)	465 (360–980)	520 (380–1022)	0.4
CT Hounsfield Unit	1054.3 ± 377.4	1136.6 ± 292.7	0.2
Laterality			0.27
Right	15 (41.7%)	27 (55.1%)	
Left	20 (55.6%)	22 (44.9%)	
Bilateral	1 (2.8%)	0	
Stone number			0.8
Single	6 (16.7%)	8 (16.3%)	
Multiple	24 (66.7%)	30 (61.2%)	
Staghorn	6 (16.7%)	11 (22.4%)	
Guy’s stone score			0.5
I	6 (16.7%)	8 (16.3%)	
II	12 (33.3%)	20 (40.8%)	
III	12 (33.3%)	10 (20.4%)	
IV	6 (16.7%)	11 (22.4%)

**Table 3.** Intraoperative and postoperative characteristics of the 85 patients treated as either day care or in-patient PCNL between January 2020 and December 2022.

	Day care PCNL	In-patient PCNL	P value
Type of anesthesia			0.6
General	31 (86.1%)	44 (89.8%)	
Spinal	5 (13.9%)	5 (10.2%)	
Tract size (median, range)	24 (13–30)	24 (18–30)	0.8
Auxiliary procedures (ECIRS)			0.08
No	29 (80.6%)	31 (63.3%)	
Yes	7 (19.4%)	18 (36.7%)	
Operative time (mean± SD)	104.8 ± 37.4	120.9 ± 38.9	0.05
Intraoperative blood loss (ml, median, IQR)	100 (50–200)	200 (100–300)	0.1
Hospital stay (hours, median, IQR)	20.4 (18.7–23.2)	49 (43.6–71)	<0.0001
Type of urinary drainage			<0.0001
Double J stent alone	32 (88.8%)	18 (37.5%)	
Nephrostomy tube + double J stent	4 (11.2%)	30 (62.5%)	
Postoperative complication rate	5 (13.8%)	11 (22.4%)	0.08
Hemoglobin deficit (median) (gm/dl)	0.8 (0.2–3)	1.2 (0.2–4)	0.3
Readmission rate	2 (5.5%)	0	0.2

A total of 6 complications occurred in 5 patients in the day care group (13.8%), all of them were minor (Clavien grade I, II) apart from a single patient presented with slipped double J stent and hematuria, obstructing the ipsilateral ureter and was readmitted to the hospital and managed by refixation of the stent (Clavien grade IIIa). Otherwise, the postoperative complication rate was comparable between both groups (13.8% vs 22.4% for day care vs in-patient group, respectively, *p* = 0.08) (Table 4).

**Table 4.** Postoperative complications that developed among the 85 patients treated as either day care or in-patient PCNL between January 2020 and December 2022.

		Complications No (%)		
Complication	Clavien Grade	Day care PCNL	In-patient PCNL	P value
Fever (>38.5 c)	Grade I	1 (2.7%)	6 (12.2%)	0.4
Urine leakage	Grade I	2 (5.5%)	1 (1.8%)	0.3
Vomiting/Ileus	Grade I	0	2 (4%)	0.2
Pyelonephritis	Grade II	1 (2.7%)	0	0.2
Bleeding/hematuria (requiring blood transfusion)	Grade II	1 (2.7%)	2 (4%)	0.3
Slipped double J stent & obstructive uropathy (blood clots)	Grade IIIa	1 (2.7%)	0	0.2

## Discussion

Despite the trend of performing surgical procedures as day case as far as possible, PCNL in most institutions is still carried out on in-patient basis, mainly because of patient safety purposes. Complications during the surgery or unwanted postoperative events, like injury to the collecting system, bleeding, sepsis, and pain are the main concerns necessitating hospital admission [9]. There is a growing tendency worldwide to encourage early hospital discharge. Day care surgeries allow early ambulation, earlier return to daily activities, and decrease the risk of nosocomial infection. Last but not the least, day care operation also reduces the cost and results in better utilization of the hospital resources, which is another important issue after patient safety [11,12].

Several publications reporting the outcomes of day care- or ambulatory PCNL have proved that day care PCNL is safe and effective [10–18]. In a systematic review, age more than 18, living close to the hospital or having quick access to the health care facility, motivated and compliant patient with adequate family support and social status, normal renal function and contralateral kidney, ASA I-II and BMI < 30 and a single stone were recommended for ambulatory PCNL. It was not recommended in obese patients with BMI > 30, having multiple comorbidities, active cardiac disease, presence of large stone burden as partial or complete staghorn stone, and in special anatomical situations like solitary or transplant kidney, encrusted double J stent or congenital malformations [10].

Our current study did not focus on the postoperative stone-free rate because we did not find any correlation between the initial stone burden and the possibility of early discharge. Nevertheless, in a meta-analysis published in 2020, a comparison was carried out between a day care and an in-patient PCNL group. There was no significant difference in the stone-free rate and the complication rate, and there was no significant difference in terms of the postoperative readmission rate either [16].

In one of the latest publications from April 2022, the authors published their experience and results with ambulatory PCNL with extended patient selection criteria [17]. Of their 118 patients involved in the study, 92 (78%) met extended criteria. According to their results, day care PCNL could not only be performed successfully and safely in the presence of the previously recommended inclusion criteria but they extended the patient selection with solitary kidney, transplant kidney and creating multiple tracts. In the same study, the readmission rate was 5%, which varied between 1.4% and 10% in previous publications [16,18].

The feasibility of a successfully and safely carried out day care PCNL is based on the establishment of strict patient inclusion criteria, and the readiness of an advanced health care facility the procedure is being performed in. Recent studies reported that ambulatory PCNL can be carried out nearly 100% of the cases and suggested that this procedure can safely be performed in centers with sufficient experience and case volume [19–21]. Once the intervention is carried out safely with the previously discussed conditions and criteria and with gaining more and more experience and evidence, the inclusion criteria might be carefully expanded with patients with higher stone burden including staghorn stones, more complex, however stable comorbidities, or obesity. We already published a case report in 2021 about such patient underwent tubeless supine day care PCNL under spinal anesthesia [22].

In our study, we analyzed our initial experience with PCNL on a day care basis in a governmental hospital in a middle East country. We worked out our own inclusion criteria and conditions to discharge patients in safety within 24 hours after the surgery. Moreover, the interventions were carried out by multiple experienced surgeons. Numerous patients had a partial or complete staghorn stone because we had all the technical and instrumental possibilities to routinely practice flexible ureteroscopy with generous indication in terms of stone burden. Although, our patient population was basically young, being most of them without any medical disease, we could successfully discharge selected patients with high stone burden including staghorn stones, bilateral stones, overweight patients, patients with controlled medical comorbidities or undergoing spinal anesthesia.

There were only 2 readmissions (5%), which rate corresponds with the international literature [18]. Ninety five percent of our patients appeared at the OPD clinic for follow-up without any major complaints, indicating that our discharge criteria were appropriate. Unfortunately, our study was severely hampered and negatively impacted by the Covid pandemic. We were also desperately forced to delay all elective surgeries for a total of 7 months of the period reviewed, due to the second and third waves. Nevertheless, our study has the 2^nd^ highest number among the reviewed contemporary literature performing the ambulatory procedures solely in supine position, since the vast majority of the studies were carried out with prone PCNL [10,13,23]. Several limitations of this study should be acknowledged. First, our results should be interpreted in the context of a retrospective study. Small sample size and lack of randomization also represent limitations. Cost analysis of each procedure couldn’t be calculated precisely either. However, we believe that our encouraging results will motivate us and other colleagues to address these issues in future, well-designed randomized control trials.

## Conclusion

Our study supports, that with appropriate experience and practice, with well-established patient pathway protocols, and having advanced hospital facilities as well as appropriate patient safety measures, day care PCNL can be performed safely in a reasonable proportion of patients.

## Data Availability

The data that support the findings of this study are available from the corresponding author, upon reasonable request.

## Research approvals

The research was approved by the institutional review board of Hamad Medical Corporation. Approval number is SR\RE\2023\45.
